# Levels of Arachidonic Acid–Derived Oxylipins and Anandamide Are Elevated Among Military *APOE* ɛ4 Carriers With a History of Mild Traumatic Brain Injury and Post-Traumatic Stress Disorder Symptoms

**DOI:** 10.1089/neur.2023.0045

**Published:** 2023-09-25

**Authors:** Aurore Nkiliza, Claire J.C. Huguenard, Gregory J. Aldrich, Scott Ferguson, Adam Cseresznye, Teresa Darcey, James E. Evans, Michael Dretsch, Michael Mullan, Fiona Crawford, Laila Abdullah

**Affiliations:** ^1^ Roskamp Institute, Sarasota, Florida, USA.; ^2^ Open University, Milton Keynes, United Kingdom.; ^3^ James A. Haley VA Hospital, Tampa, Florida, USA.; ^4^ U.S. Army Medical Research Directorate-West, Walter Reed Army Institute of Research, Joint Base Lewis-McChord, Washington, USA.; ^5^ U.S. Army Aeromedical Research Laboratory, Fort Novosel, Alabama, USA.

**Keywords:** apolipoprotein E (APOE), arachidonic acid (AA), ethanolamides, mild TBI, oxylipins

## Abstract

Currently approved blood biomarkers detect intracranial lesions in adult patients with mild to moderate traumatic brain injury (TBI) acutely post-injury. However, blood biomarkers are still needed to help with a differential diagnosis of mild TBI (mTBI) and post-traumatic stress disorder (PTSD) at chronic post-injury time points. Owing to the association between phospholipid (PL) dysfunction and chronic consequences of TBI, we hypothesized that examining bioactive PL metabolites (oxylipins and ethanolamides) would help identify long-term lipid changes associated with mTBI and PTSD. Lipid extracts of plasma from active-duty soldiers deployed to the Iraq/Afghanistan wars (control = 52, mTBI = 21, PTSD = 34, and TBI + PTSD = 13) were subjected to liquid chromatography/mass spectrometry analysis to examine oxylipins and ethanolamides. Linear regression analyses followed by *post hoc* comparisons were performed to assess the association of these lipids with diagnostic classifications. Significant differences were found in oxylipins derived from arachidonic acid (AA) between controls and mTBI, PTSD, and mTBI + PTSD groups. Levels of AA-derived oxylipins through the cytochrome P450 pathways and anandamide were significantly elevated among mTBI + PTSD patients who were carriers of the apolipoprotein E E4 allele. These studies demonstrate that AA-derived oxylipins and anandamide may be unique blood biomarkers of PTSD and mTBI + PTSD. Further, these AA metabolites may be indicative of an underlying inflammatory process that warrants further investigation. Future validation studies in larger cohorts are required to determine a potential application of this approach in providing a differential diagnosis of mTBI and PTSD in a clinical setting.

## Introduction

Traumatic brain injury (TBI) and post-traumatic stress disorder (PTSD) are signature wounds of the Iraq/Afghanistan wars.^1,2^ Among 28% of the service members who experience a TBI, 33–65% also develop PTSD.^3–6^ Even though 82.3% of these injuries are classified as mild TBI (mTBI), a proportion of mTBI patients experience chronic symptoms.^7^ mTBI is caused by a direct impact on the brain, resulting in biochemical changes that correspond with altered states of consciousness,^8–10^ whereas neurobiological changes in PTSD are associated with witnessing a traumatic event.^11^ Despite the differences in etiologies of PTSD and TBI, there is a substantial overlap of symptoms, including changes in cognition and psychological health.^4,12,13^ Both mTBI and PTSD also share several pathophysiological features such as neuroinflammation, oxidative stress, and excitotoxicity.^13^ As such, there remains a need for objective blood biomarkers that can be utilized routinely, both in the field and bedside settings, to help with a differential clinical diagnosis of mTBI and PTSD.^14,15^

Transport of proteins, lipids, and solutes in and out of the brain is regulated by the blood–brain barrier (BBB).^16^ After severe brain injuries, a disruption of the BBB allows the leakage of brain proteins and other factors into the periphery.^17,18^ In the absence of an overt BBB disruption in mTBI, transfer of brain products into the periphery could occur by a passive efflux of brain-derived material into the cerebrospinal fluid (CSF) through the glymphatic or exosomal transports into the periphery.^19–21^ In 2018, the combination of glial fibrillary acidic protein (GFAP) and ubiquitin carboxyl-terminal hydrolase-L1 (UCH-L1) was approved by the U.S. Food and Drug Administration as a blood test for detecting intracranial lesions in mild/moderate TBI adult patients within 12 h of injury.^22^ Recently, GFAP and UCH-L1 were shown to distinguish mTBI and non-TBI trauma^23^ and identify mTBI patients with computed tomography abnormalities.^24^ However, the utility of these blood biomarkers for a differential diagnosis of chronic mTBI and PTSD remains unknown.^25–29^

Studies show that TBI-triggered brain lipid changes are reflected in the blood, suggesting that blood lipids could be used as biomarkers for TBI.^30–33^ Among brain lipids, n-6 arachidonic acid (AA; 20:4n-6), linoleic acid (LA; 18:2n-6), and n-3 docosahexaenoic acid (DHA; 22:6n-3) are polyunsaturated fatty acids (PUFAs) that represent important components of the brain membrane phospholipids (PLs).^34,35^ These PUFAs are precursors of oxylipins—metabolites synthesized by cyclooxygenase (COX), lipoxygenase (LOX), and cytochrome P450 (CYP)—that regulate multiple physiological processes within the brain, including synaptic transmission, vasodilation, neuronal morphology, blood flow, and inflammation.^36–40^ Ethanolamides are derived from PLs and are endocannabinoids shown to exert anti-inflammatory and analgesic effects.^41^

Another factor linking lipid metabolism to TBI and PTSD is the apolipoprotein E (APOE) gene, which has three major polymorphisms: E2, E3, and E4. Among these, the E4 allele is associated with a high risk of developing late-onset Alzheimer's disease (AD).^42^ Epidemiological studies have reported an association between the presence of the E4 allele among TBI patients with the risk of developing AD with age.^43,44^ The E4 allele is associated with poor functional and cognitive outcomes in both TBI and PTSD.^45,46^ An increase in AA-to-DHA ratios was found in the serum of E4^+^ pre-clinical AD patients,^47^ suggesting an imbalance toward increased production of proinflammatory lipid metabolites derived from AA.^48,49^ Evidence suggests that the LOX enzyme is dysregulated in the context of AD, and there is a reduced expression of 15-LOX in the hippocampus of post-mortem AD patients.^50^ As such, E4-associated changes in blood oxylipins in TBI could indicate underlying neurodegenerative processes after inflammatory and oxidative stress.

We hypothesized that bioactive lipid metabolites of PUFAs would be elevated in mTBI, PTSD, and mTBI + PTSD groups compared to healthy controls. Hence, the objective of this exploratory study was to characterize whether plasma oxylipins and ethanolamides differ between controls, mTBI, PTSD, and mTBI + PTSD diagnostic groups using a cohort of active-duty military soldiers returning to combat zones in the Middle East.

## Methods

### Study participants

**FIG. 1.**
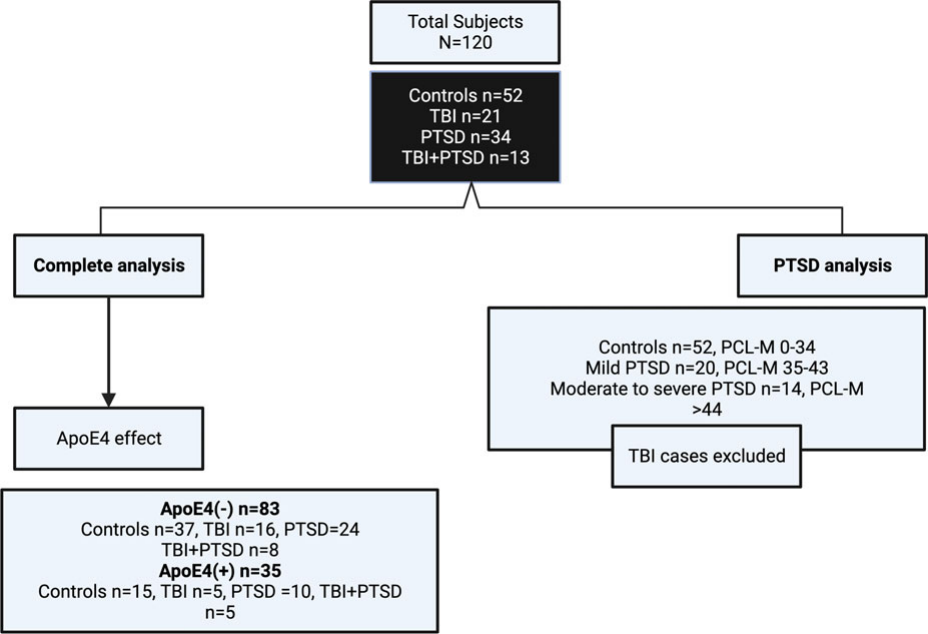
Flowchart of 120 volunteer soldiers who participated in the study. Flowchart shows the breakdown of 120 participants and their diagnostic classifications, which are then further stratified by APOE E4 allele carrier status. APOE, apolipoprotein E; PCL-M, PTSD Checklist–Military Version; PTSD, post-traumatic stress disorder; TBI, traumatic brain injury.

Samples from a published cohort evaluating major lipid profiles and genetic contributions were used for the current study.^51,52^ The study was approved by brigade commanders and by the institutional review board at Headquarters U.S. Army Medical Research and Materiel Command. In 2010 and 2011, volunteering soldiers from two brigade combat teams were recruited and enrolled at a designated military installation ∼30 days before a 12-month deployment to the Middle East. There were no inclusion/exclusion criteria given that participation was voluntary, and all soldiers were deemed medically fit for deployment (see Fig. 1 for the flowchart).^51^ Screening for mTBI was performed with the Defense and Veterans Brain Injury Center–Brief Traumatic Brain Injury Screen.^53^ Classification of mTBI required both the endorsement of an injury-related event and an altered state of consciousness.^51,52^ A classification of PTSD was based on a score ≥35 on the PTSD Checklist–Military Version (PCL-M).^54^ Participants were considered controls if they reported no history of PTSD, TBI, and depression and were negative for both PTSD and depression screens.^51^ Post-concussive symptoms were assessed using the Neurobehavioral Symptom Inventory (NSI); depression, alcohol dependency, anxiety, stress level, sleep quality, and daytime sleepiness were also assessed.^55–59^ Neurocognitive functioning was assessed using Central Nervous System–Vital Signs.^60^ Non-fasting pre-coded whole blood was collected in ethylenediamine tetraacetic acid tubes and processed on-site by study staff blinded to participants' diagnoses. Whole blood was centrifuged at room temperature at 1380*g* for 5 min to collect plasma, which was aliquoted in 1.5-mL Eppendorf tubes and then shipped on dry ice and stored frozen in −80°C freezers upon arrival until experimentation.^52,61^ The lipid experiments below were performed between 2019 and 2020.

### Ethanolamide assay

Plasma (100 μL) was spiked with 5 μL of ethanolamide internal standard (IS) mix (see Supplementary Methods Table S1). Three volumes of methanol (MeOH) were combined with samples and centrifuged at 10,800 revolutions per minute (RPM) for 10 min at 4°C. Approximately 70% of the supernatant was collected before adding 160 μL of H_2_O. For solid phase extractions (SPEs), cartridges (Oasis PRiME HLB 1 cc Vac Cartridge, 30 mg Sorbent; Waters Corporation, Milford, MA) were pre-conditioned as per the manufacturer's instructions, loaded with the samples in 1 mL of 5% MeOH, and dried for 5 min. Ethanolamides were eluted by applying 500 μL of 90:10 acetonitrile (ACN)/MeOH (v/v%) to the cartridges, then vacuum dried and reconstituted in 50 μL of 50% ACN for filtering with 0.2-μm centrifuge filters (ThermoFisherScientific, Waltham, MA), centrifuged at 10,800 RPM for 10 min at 4°C, and subjected to reversed-phase liquid chromatography (LC)/mass spectrometry analyses.

Separation of ethanolamides was performed with a Thermo Scientific™ UltiMate™ 3000 LC system using a Kinetex 2.6-μm XB-C18 100 Å, 100 × 1.0 mm column (Phenomenex, Torrance, CA), where solvent A contained 10% ACN and solvent B consisted of MeOH with an addition of 5 mM of ammonium acetate and 0.1% acetic acid as a modifier in both mobile phases. Separation was achieved within 7 min under isocratic conditions at 90% B throughout the run. Full-scan fragmentation spectra of analytes were acquired using parallel reaction monitoring at 17,500 resolution, automatic gain control (AGC) target was set to 2e5, maximum injection time (max IT) of 100 ms, and an isolation window of 2 m/z. Normalized collision energies were optimized for each species (Supplementary Table S1).

### Oxylipin assay

Plasma (250 μL) was spiked with 5 μL of 10 mg/mL of butylated hydroxytoluene and 5 μL of eicosanoid IS mix (see Supplementary Table S1). The quality control (QC) sample was spiked with 5 μL of an unlabeled standard mix containing all target analytes (see Supplementary Table S2) at a concentration of 1 μg/mL, then 750 μL of ice-cold MeOH +2% formic acid (FA) was added to the samples, then centrifuged at 10,800 RPM at 4°C for 10 min, and supernatant was collected followed by SPE cleanup as above. Flow-through was collected, combined with 500 μL of H_2_O + 2% FA, reloaded twice, washed with 500 μL of 5% MeOH +2% FA, and dried completely. Analytes were eluted with 500 μL of 90:10 ACN/MeOH (v/v%) + 2% FA in 16 μL of 30% glycerol, dried under a gentle stream of nitrogen, and reconstituted in 100 μL of 40% ACN +2% FA and filtered as above. Isolated oxylipins were separated by reversed-phase LC on the UltiMate™ 3000 LC using a Kinetex 2.6-μm XB-C18 100 Å, 100 × 1.0 mm column (Phenomenex), where solvent A contained 5% ACN and solvent B 95% ACN with an addition of 0.1% of acetic acid as a modifier in each mobile phase. The flow rate was 100 μL/min, and, for the gradient, mobile phase composition started at 45% B, increased to 55% B for 11 min, and re-equilibrated for 4 min at 45% B.

Full-scan fragmentation spectra of analytes were acquired using parallel reaction monitoring at 17,500 resolution, AGC target was set to 5e5, with max IT time of 200 ms and isolation window of 1.3 m/z (see Supplementary Table S2 for the inclusion list).

### Data processing and statistical analysis

Peak areas were integrated using Tracefinder^TM^ software, and a mass window of 5 ppm was used for all ion plots. Concentrations were calculated relative to IS concentrations and normalized using a QC sample. Each sample was injected in triplicate and those with a coefficient of variance >20% were excluded from analysis. SPSS software (SPSS, Inc., Chicago, IL) and MetaboAnalyst 5.0 were used for statistical analysis. Kendall's tau-b correlations were performed on the data. Data were normalized, scaled, and analyzed with Ward's clustering method, and the top analytes were selected by analysis of variance (ANOVA). Heatmaps were generated using *z*-transformed variables, and significance testing of each analyte was performed using mixed linear modeling (MLM) to examine the independent effects of APOE and diagnosis as fixed factors on lipid outcomes (dependent variables), with a diagonal covariance matrix and repeated measurements incorporating technical replicates as a random factor to account for random noise in the data sets.^62^ A Benjamini-Hochberg (BH) correction was performed on all multiple comparisons after MLM analyses.^52^ For non-normally distributed data, the Kruskal-Wallis test was applied. The *p*-value threshold for significance was a false discovery rate <0.1.

## Results

### Ethanolamides and oxylipins correlate within lipid classes

**FIG. 2.**
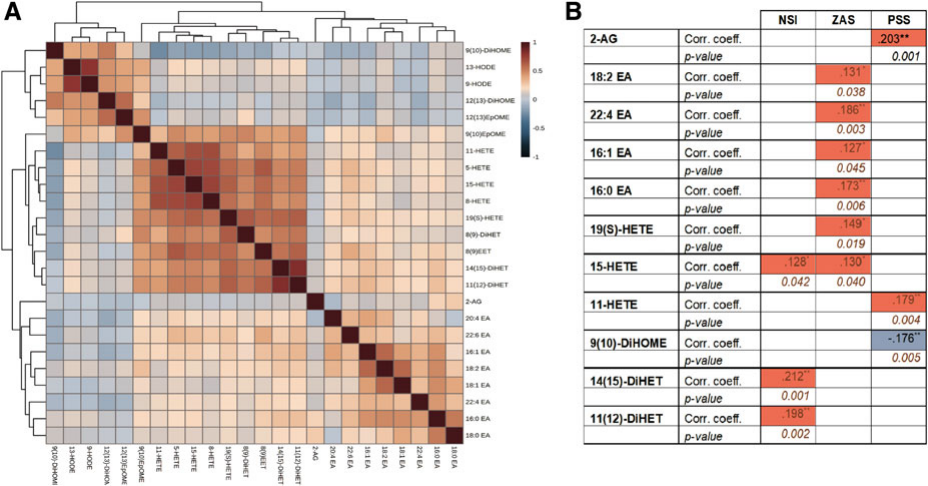
Plasma oxylipins and ethanolamide correlations. (**A**) Heatmap showing correlation coefficients. (**B**) Table showing significant correlations between self-report measures, ethanolamides, and oxylipins. Statistics: Kendall's tau-b correlations. corr. coeff., correlation coefficient; NSI, Neurobehavioral Symptoms Inventory; PSS, Perceived Stress Scale; ZAS, Zung Self-Rating Anxiety Scale.

All participants in this study were males (controls = 52, PTSD = 34, mTBI = 21, and mTBI + PTSD = 13). There were no significant differences in allelic distribution of APOE, age, race, education, or previous number of deployments between diagnostic groups with and without E4 stratification (Table 1). Based on self-report, 16 controls, 7 TBI, and 11 PTSD participants reported that the current deployment was their first deployment. Five TBI and 9 TBI + PTSD participants reported experiencing brain damage in the past year. Several lipids (DiHOME, EpOMEs, and HODEs) had a positive correlation with each other (cluster 1; Fig. 2A). Additionally, HETEs, DiHETEs, and EETs were positively correlated with each other (cluster 2; Fig. 2A).

**Table 1.** Basic Demographics of the Study Population

		Control *n* = 52		mTBI *n* = 21		PTSD *n* = 34		mTBI+PTSD *n* = 13	
***APOE* E4**		-	+	-	+	-	+	-	+
**Numbers**		*n* = 37	*n* = 15	*n* = 16	*n* = 5	*n* = 24	*n* = 10	*n* = 8	*n* = 5
**Age** (mean ± SD)		27 ± 7	27 ± 8	25 ± 4	30 ± 11	28 ± 8	23 ± 4	30 ± 7	29 ± 4
**Education** (mean ± SD)		13 ± 1	13 ± 1	13 ± 1	13 ± 2	13 ± 1	13 ± 1	14 ± 2	14 ± 1
**Race** (*n*)	Black	1	0	1	1	2	2	0	0
	White	29	10	11	4	18	7	7	3
	Pacific Islander	2	0	1	0	0	1	1	0
	Hispanic/Latino	4	1	2	0	3	0	0	2
	Native American	1	0	0	0	0	0	0	0
	Asian	0	1	0	0	1	0	0	0
	Other	0	2	1	0	0	0	0	0
**First deployment (Yes)**		11	5	5	2	6	5	0	0
**TBI and PTSD**	No reported TBI events	35	14	0	0	0	0	0	0
	Knockout without concussion	2	1	0	0	0	0	0	0
	Concussion with LOC	0	0	6	3	5	5	7	4
**Reported brain damage in prior year (Yes)**		0	0	5	0	3	6	5	4
**Diagnosed with PTSD ever in life (Yes)**		0	0	1	0	24	10	8	5
**Total number of deployments**	0	11	6	5	2	6	5	0	0
	1	15	6	5	0	12	3	2	1
	2+	11	3	6	3	6	2	6	4
**Medication use** (*n*)	None	33	14	15	5	14	10	5	4
	Anti-depressants	0	0	1	0	**5**	0	1	0
	Anti-inflammatories	1	1	0	0	1	0	1	1
	Analgesics	0	0	0	0	1	0	1	0
	Sedatives & hypnotics	0	0	1	0	0	0	1	0
	Anti-bacterial	1	0	0	0	1	0	0	0
	Cardiovascular medications	2	0	0	0	3	0	0	0
	Gastrointestinal agents	1	0	0	0	2	0	0	0
	Allergy medication	0	1	0	0	0	0	0	0
	Headaches/migraines	0	0	1	0	1	0	0	1
**NSI** (mean ± SD)		5 ± 7	7 ± 7	13 ± 14	10 ± 6	30 ± 15	24 ± 15	26 ± 21	25 ± 12
**ZAS** (mean ± SD)		30 ± 5	29 ± 6	**33 ± 11**	31 ± 4	**38 ± 10**	**37 ± 9**	**36 ± 8**	31 ± 8
**PSS** (mean ± SD)		28 ± 13	27 ± 15	28 ± 13	26 ± 11	29 ± 8	29 ± 8	28 ± 9	32 ± 4

Statistics: Kruskal-Wallis with B-H correction or Chi-square as appropriate. Bolded numbers indicate significant differences between groups against their respective controls. For medication use, 5 participants on multiple medications. Abbreviations; mTBI: mild traumatic brain injury, PTSD, post-traumatic stress disorder; NSI, Neurobehavioral Symptoms Inventory; ZAS, Zung Self-Rating Anxiety Scale; PSS: Perceived Stress Scale.

There were significant associations between self-report measures on psychological health (Zung Self-Rating Anxiety Scale [ZAS] and Perceived Stress Scale [PSS]) and post-concussive symptoms (NSI) with ethanolamides and oxylipin species (Fig. 2B). Specifically, NSI scores were positively associated with plasma 15-HETE and two DiHET species. There was a positive association between PSS scores and plasma HETE species and 2-AG, but a negative association with 9(10)-DiHOME levels (Fig. 2B).

### Subclasses of bioactive lipid metabolites are differentially affected in mTBI, PTSD, and mTBI + PTSD diagnoses

**FIG. 3.**
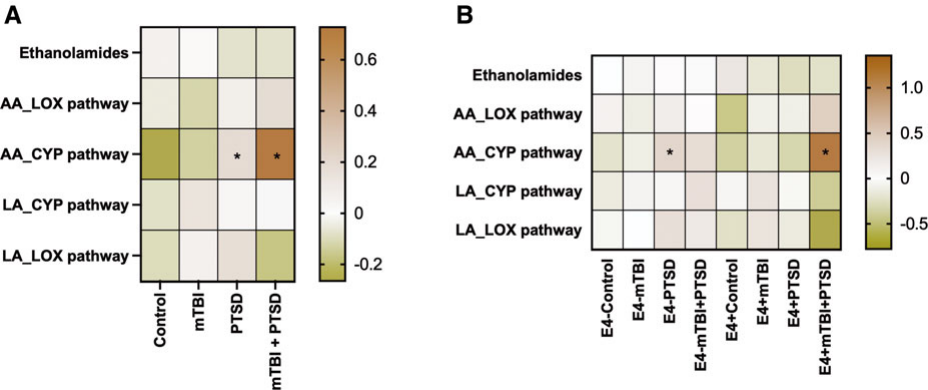
Plasma levels of grouped AA- and LA-derived oxylipins. Heatmap shows the average z-score of all persons within each diagnosis group regarding their E4 status. Statistics: one-way ANOVA and LSD *post hoc* comparison followed by BH correction (*BH-corrected *p* < 0.1). Black asterisks represent significant differences between diagnosis groups and their respective controls within the same genotype group. AA, arachidonic acid; ANOVA, analysis of variance; CYP, cytochrome P450; BH, Benjamini-Hochberg; LA, linoleic acid; LOX, lipoxygenase; LSD, least significant difference; mTBI, mild traumatic brain injury; PTSD, post-traumatic stress disorder.

**FIG. 4.**
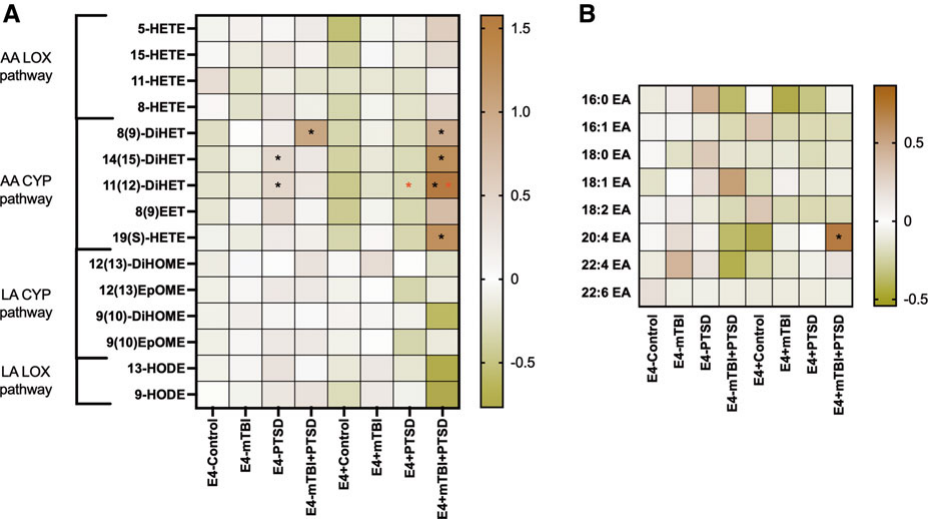
Plasma levels of oxylipins produced by LOX and CYP pathways (left) and ethanolamides (right). Heatmap shows the average *z*-score of all persons within each diagnosis group regarding of their e4 status. Statistics: one-way ANOVA and LSD *post hoc* comparison followed by BH correction (*BH-corrected *p* < 0.1). Black asterisks represent significant differences between diagnosis groups and their respective controls within the same genotype group. Red asterisks represent significant differences between non-e4 carriers (e4^–^) and e4 carriers (e4^+^) within the same diagnosis group. AA, arachidonic acid; ANOVA, analysis of variance; CYP, cytochrome P450; BH, Benjamini-Hochberg; EA, ethanolamides; LA, linoleic acid; LOX, lipoxygenase; LSD, least significant difference; mTBI, mild traumatic brain injury; PTSD, post-traumatic stress disorder.

To examine the class effects of AA and LA metabolites derived from different enzymatic pathways, we grouped the oxylipin species based on their biosynthetic pathways (Fig. 3). These studies showed that AA-derived oxylipins generated by CYP pathways were elevated among participants with PTSD and mTBI + PTSD (BH-corrected *p* < 0.05; Fig. 3A). Among non-E4 carriers, these oxylipins were significantly increased in the PTSD group compared to controls (Fig. 3B). The same oxylipin subgroup was also increased in the E4^+^ mTBI + PTSD group versus the E4^+^ controls (BH-corrected *p* < 0.1; Fig. 3B)**.** Non-E4 carriers showed that 14(15)-DiHET and 11(12)-DiHET were increased in the PTSD group, whereas 8(9)-DiHET was increased in the mTBI + PTSD group compared to the control group. Among E4 carriers, AA oxylipins derived through the CYP pathway, except for 8(9) EET, were significantly increased in the mTBI + PTSD group compared to the control group (Fig. 4A). E4 carriers with mTBI + PTSD and PTSD alone had a higher level of plasma 11(12)-DiHET than non-E4 carriers with the same diagnosis (Fig. 4A). Among E4 carriers, levels of 20:4EA (anandamide) were increased in mTBI + PTSD compared to controls (Fig. 4B).

### *Oxylipins are increased in mTBI + PTSD and further modulated by* APOE *E4*

**FIG. 5.**
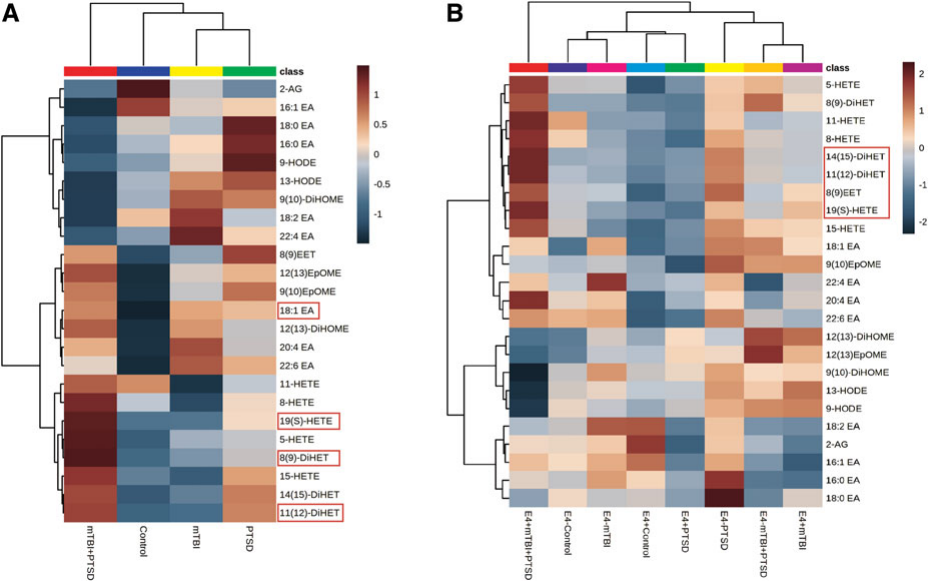
Plasma oxylipins and ethanolamide are altered with mTBI and PTSD diagnosis, particularly among *APOE* E4 carriers. (**A**) Hierarchical clustering heatmap showing ethanolamide and oxylipin species profiles in diagnostic groups. (**B**) Hierarchical clustering heatmap showing ethanolamide and oxylipin species profiles in diagnostic groups stratified by *APOE* E4 carrier status. Statistics: normalized and scaled data with Ward's method. Analytes significantly altered between groups (*p* < 0.05) are indicated in red. mTBI, mild traumatic brain injury; PTSD, post-traumatic stress disorder.

Hierarchical clustering was performed for determining their inter-relationship with each other (Fig. 5A). Compared to controls, persons with mTBI + PTSD had a clustering of many ethanolamide species. HODE and di-HOME were reduced, whereas most other clusters were increased, including AA and DHA containing ethanolamide species (20:4EA and 22:6EA, respectively). Opposite trends for clustered oxylipins and EA were observed for mTBI and PTSD compared to controls. The effect of diagnosis on ethanolamide and oxylipin profiles was modulated by E4 carrier status (Fig. 5B), with the E4^+^ mTBI + PTSD group showing strong clustering effects on HETE, DiHET, and EET species. The influence of non-E4 status on differential clustering of oxylipins and ethanolamides was noted for controls compared to PTSD groups, whereas E4 influence on mTBI compared to controls was further apart from each other (Fig. 5B). See Supplementary Data Table S3 for concentrations.

## Discussion

Blood biomarkers are needed to help with the differential diagnoses of mTBI and PTSD in civilian, active-duty, and veteran populations.^14,15^ Currently, approved blood biomarkers are useful for detecting intracranial lesions in mild-to-moderate TBI adult patients within hours post-injury. However, diagnosing mTBI at subacute and chronic post-injury time points remains challenging.^63–65^ There are also no approved biomarkers for PTSD.^66,67^ Hence, there remains a need for reliable, low-cost, and selective biomarkers for providing a differential diagnosis of mTBI and PTSD given their clinical similarities, frequent comorbidity, and the lack of availability of reliable markers that would allow discrimination between these two conditions.

Blood lipids may indicate the underlying inflammatory process in the brain^52,61,68,69^ and many oxidized PUFAs are implicated in vascular injuries, as in the case of ischemic injuries, and could reflect ongoing secondary vascular dysfunction associated with BBB damage.^70^ Past profiling of plasma lipids in this cohort suggests alterations of lipid metabolism and homeostasis in comorbid mTBI and PTSD,^52,61^ suggesting that these lipids may help with a differential classification of these two conditions when they are comorbid with each other.

Given their known role in inflammation, the current study examined bioactive lipid metabolites, ethanolamides and oxylipins, that are derived from PL. Changes in these lipid metabolites have been reported in non-alcoholic fatty liver disease, obesity, type 2 diabetes, and several cardiovascular diseases for which chronic inflammation is a major contributor.^71^ Because oxylipins are generated by the oxidation of different PUFAs through COX, LOX, and CYP pathways, we analyzed oxylipin profiles stratified by oxylipin's precursors and synthesis pathways. Levels of AA-derived oxylipins synthesized by the CYP pathway are differentially modulated by PTSD and mTBI + PTSD diagnoses. Many of these species were associated with PTSD and mTBI symptoms. Vasculature damage in TBI is often followed by increased vasodilatation contributing to TBI-related swelling of the brain, whereas vasoconstriction, attributable to psychological stress, is a characteristic of PTSD.^72–74^ There is increasing evidence that inflammation contributes to clinical and functional outcomes of TBI.^74^ In PTSD, an increase in proinflammatory and a reduction in anti-inflammatory cytokines has also been reported.^75^ Inhibition of COX and LOX pathways post-injury reduces inflammation, suggesting that immune responses to injury are significantly affected by HETEs and AA-derived prostanoids.^76–78^ An increase of 10-fold in 5-HETE and a 17-fold increase in 12-HETE were noted in CSF from TBI patients compared to controls.^79^ Improved recovery from TBI was associated with greater concentrations of 13-HODE.^80^

Past studies have suggested that with aging, there is increased oxidation of PUFAs, particularly among E4 carriers.^81,82^ Our current studies suggest that among patients with mTBI + PTSD, oxidized PUFAs are significantly elevated compared to all other groups. Though the molecular mechanism of such an association remains to be determined, these findings suggest that factors other than aging and neurodegeneration could increase PUFA oxidation in the presence of the E4 allele. These oxidized PUFAs could serve as differential markers of comorbidity of mTBI and PTSD. Future studies are required to better understand the role of these lipids in the long-term chronic sequelae of mTBI and PTSD. Limitations of the study include a small sample size in each of the diagnostic categories, the predominantly white and male composition of the cohort, and recall bias associated with self-report of time and type of injury. Given that most participants had been deployed before and reported experiencing brain damage in the past year, mTBI will likely be representative of chronic injury. As such, future studies are required to better understand the role of these lipids in the long-term chronic sequelae of mTBI and PTSD.

## Conclusion

This study demonstrates that peripheral oxylipins may serve as a potential source of biomarkers to differentiate persons suffering from the consequences of mTBI from those with PTSD. Though some limitations minimize the generalization of these findings, there is an internal consistency of biological responses in the presence of the E4 allele, which suggests that oxidation of PUFAs in E4 is a mediator of secondary inflammation and vascular pathologies associated with mTBI and PTSD. Given that there are currently no reliable biomarkers for detecting comorbid mTBI and PTSD, as well as differentiating mTBI from PTSD, analyses of plasma oxylipins could serve to develop low-cost, easily accessible, and minimally invasive alternatives in the identification of mTBI and PTSD biomarkers.

## Supplementary Material

Supplemental data

## Data Availability

Raw data are available upon a written request.

## Abbreviations Used

AA: arachidonic acid
ACN: acetonitrile
AD: Alzheimer's disease
AGC: automatic gain control
ANOVA: analysis of variance
APOE: apolipoprotein E
BBB: blood–brain barrier
BH: Benjamini-Hochberg
COX: cyclooxygenase
CSF: cerebrospinal fluid
CYP: cytochrome P450
DHA: docosahexaenoic acid
FA: formic acid
GFAP: glial fibrillary acidic protein
IS: internal standard
LA: linoleic acid
LC: liquid chromatography
LOX: lipoxygenase
max IT: maximum injection time
MeOH: methanol
MLM: mixed linear modeling
MS: mass spectrometry
mTBI: mild TBI
NSI: Neurobehavioral Symptom Inventory
PCL-M: PTSD Checklist–Military Version
PL: phospholipid
PSS: Perceived Stress Scale
PTSD: post-traumatic stress disorder
PUFAs: polyunsaturated fatty acids
QC: quality control
RPM: revolutions per minute
TBI: traumatic brain injury
UCH-L1: ubiquitin carboxyl-terminal hydrolase-L1
ZAS: Zung Self-Rating Anxiety Scale
