# Supplementary material for: Levels of Arachidonic Acid–Derived Oxylipins and Anandamide Are Elevated Among Military APOE ɛ4 Carriers With a History of Mild Traumatic Brain Injury and Post-Traumatic Stress Disorder Symptoms
Source: Neurotrauma Rep. 2023 Sep 25;4(1):643–54. doi: 10.1089/neur.2023.0045 (PMC10541938; doi:10.1089/neur.2023.0045)
Supplement: Supplementary file 1 [file Supp_DataMethods.odt]

Supplementary Table S1. Ethanolamide assay inclusion list. Abbreviation; IS: internal standard, (N)CE: (normalized) collision energy.

| Compound | Abbreviation | Formula | Compound type | Precursor (m/z) | (N)CE | Product (m/z) | IS used for quantitation |
| --- | --- | --- | --- | --- | --- | --- | --- |
| Palmitoyl ethanolamide-d4 | 16:0 EA-d4 | C18H33 D4NO2 | IS | 304.315 | 28 | 62.061 | N/A |
| Stearoyl ethanolamide-d3 | 18:0 EA-d3 | C20H38 D3NO2 | IS | 331.340 | 28 | 62.061 | N/A |
| Oleyl ethanolamide-d2 | 18:1 EA-d2 | C20H37D2NO2 | IS | 328.318 | 28 | 62.061 | N/A |
| α-linolenoyl ethanolamide-d4 | 18:3 EA-d4 | C20H31 D4NO2 | IS | 326.299 | 28 | 66.09 | N/A |
| Arachidonoyl ethanolamide-d8 | 20:4 EA-d8 | C22H29 D8NO2 | IS | 356.340 | 28 | 62.061 | N/A |
| Docosahexaenoyl ethanolamide-d4 | 22:6 EA-d4 | C24H33 D4NO2 | IS | 376.315 | 28 | 66.09 | N/A |
| 2-Arachidonoylglycerol-d5 | 2-AG-d5 | C23H33 D5O4 | IS | 384.216 | 25 | 287.24 | N/A |
| Palmitoyl ethanolamide | 16:0 EA | C18H37NO2 | Target | 300.290 | 28 | 62.061 | 16:0 EA-d4 |
| Palmitoleoyl ethanolamide | 16:1 EA | C18H35NO2 | Target | 298.274 | 28 | 62.061 | 18:3 EA-d4 |
| Stearoyl ethanolamide | 18:0 EA | C20H41NO2 | Target | 328.321 | 28 | 62.061 | 18:0 EA-d3 |
| Oleoyl ethanolamide | 18:1 EA | C20H39NO2 | Target | 326.305 | 28 | 62.061 | 18:1 EA-d2 |
| Linoleoyl ethanolamide | 18:2 EA | C20H37NO2 | Target | 324.290 | 28 | 62.061 | 18:3 EA-d4 |
| Arachidonoyl ethanolamide | 20:4 EA | C22H37NO2 | Target | 348.290 | 28 | 62.061 | 20:4 EA-d8 |
| Docosatetraenoyl ethanolamide | 22:4 EA | C24H41NO2 | Target | 376.321 | 28 | 62.061 | 18:1 EA-d2 |
| Docosahexaenoyl ethanolamide | 22:6 EA | C24H37NO2 | Target | 372.290 | 28 | 62.061 | 22:6 EA-d4 |
| 2-Arachidonoylglycerol | 2-AG | C23H38O4 | Target | 379.284 | 25 | 287.24 | 2-AG-d5 |

Supplementary Table S2. Oxylipin assay inclusion list. Abbreviation; IS: internal standard, (N)CE: (normalized) collision energy.

| Compound | Abbreviation | Formula | Compound type | Precursor (m/z) | (N)CE | Products (m/z) | IS used for quantitation |
| --- | --- | --- | --- | --- | --- | --- | --- |
| (±)8,9-epoxy-5Z,11Z,14Z-eicosatrienoic acid | 8(9)EET | C20H32O3 | Target | 319.227 | 16 | 123.08, 127.075, 155.07, 167.07, 257.227 | 11(12)EET-d11 |
| (±)11,(12)-epoxy-5Z,8Z,14Z-eicosatrienoic acid-d11 | 11(12)EET-d11 | C20H21D11O3 | IS | 330.297 | 16 | 167.107, 179.107, 268.297, 312.286, 330.297 | N/A |
| 12,13-dihydroxy-9Z-octadecenoic acid | 12(13)-DiHOME | C18H34O4 | Target | 313.238 | 36 | 129.091, 183.138, 295.228 | 12(13)DiHOME-d4 |
| 12,13-dihydroxy-9Z-octadecenoic acid-d4 | 12(13)DiHOME-d4 | C18H30D4O4 | IS | 317.263 | 36 | 100.086, 131.103, 185.15, 299.252 | N/A |
| 19S-hydroxy-5Z,8Z,11Z,14Z-eicosatetraenoic acid | 19(S)-HETE | C20H32O3 | Target | 319.227 | 24 | 245.227, 289.217 | 13-HODE-d4 |
| (±)-13-hydroxy-9Z,11E-octadecadienoic acid | 13-HODE | C18H32O3 | Target | 295.227 | 30 | 195.138 | 13-HODE-d4 |
| (±)-9-hydroxy-10E,12Z-octadecadienoic acid | 9-HODE | C18H32O3 | Target | 295.227 | 30 | 171.101 | 13-HODE-d4 |
| (±)-13-hydroxy-9Z,11E-octadecadienoic acid-d4 | 13-HODE-d4 | C18H28D4O3 | IS | 299.253 | 30 | 198.157, 281.242, 299.253 | N/A |
| (±)15-hydroxy-5Z,8Z,11Z,13E-eicosatetraenoic acid | 15-HETE | C20H32O3 | Target | 319.227 | 20 | 113.096, 175.148, 219.138, 257.227, 301.217 | 15-HETE-d8 |
| (±)11-hydroxy-5Z,8Z,12E,14Z-eicosatetraenoic acid | 11-HETE | C20H32O3 | Target | 319.227 | 24 | 149.096, 167.107 | 15-HETE-d8 |
| (±)15-hydroxy-5Z,8Z,11Z,13E-eicosatetraenoic acid-d8 | 15-HETE-d8 | C20H24D8O3 | IS | 327.277 | 20 | 182.192, 226.182, 265.277, 309.267, 327.278 | N/A |
| (±)5-hydroxy-6E,8Z,11Z,14Z-eicosatetraenoic acid | 5-HETE | C20H32O3 | Target | 319.227 | 18 | 115.039, 203.18, 257.227, 301.217 | 5-HETE-d8 |
| (±)8-hydroxy-5Z,9E,11Z,14Z-eicosatetraenoic acid | 8-HETE | C20H32O3 | Target | 319.227 | 18 | 155.07, 163.07, 257.227, 301.217 | 5-HETE-d8 |
| (±)5-hydroxy-6E,8Z,11Z,14Z-eicosatetraenoic acid-d8 | 5-HETE-d8 | C20H24D8O3 | IS | 327.277 | 18 | 116.045, 210.224, 265.277, 309.267, 327.278 | N/A |
| (±)9,10-dihydroxy-12Z-octadecenoic acid | 9(10)-DiHOME | C18H34O4 | Target | 313.238 | 36 | 171.101, 201.112, 277.217 | 9(10)DiHOME-d4 |
| (±)14,15-dihydroxy-5Z,8Z,11Z-eicosatrienoic acid | 14(15)-DiHET | C20H34O4 | Target | 337.238 | 26 | 129.091, 207.138, 319.228 | 9(10)DiHOME-d4 |
| (±)11,12-dihydroxy-5Z,8Z,14Z-eicosatrienoic acid | 11(12)-DiHET | C20H34O4 | Target | 337.238 | 26 | 167.107, 197.117, 319.228 | 9(10)DiHOME-d4 |
| (±)8,9-dihydroxy-5Z,11Z,14Z-eicosatrienoic acid | 8(9)-DiHET | C20H34O4 | Target | 337.238 | 26 | 127.075, 141.091, 185.081, 319.228 | 9(10)DiHOME-d4 |
| (±)9,10-dihydroxy-12Z-octadecenoic acid-d4 | 9(10)DiHOME-d4 | C18H30D4O4 | IS | 317.263 | 36 | 172.108, 203.125, 281.242, 299.252 | N/A |
| (±)12(13)epoxy-9Z-octadecenoic acid | 12(13)EpOME | C18H32O3 | Target | 295.227 | 26 | 195.138 | 9(10)EpOME-d4 |
| (±)9,10-epoxy-12Z-octadecenoic acid | 9(10)EpOME | C18H32O3 | Target | 295.227 | 26 | 171.102 | 9(10)EpOME-d4 |
| (±)9,10-epoxy-12Z-octadecenoic acid-d4 | 9(10)EpOME-d4 | C18H28D4O3 | IS | 299.252 | 26 | 172.108, 281.242, 299.252 | N/A |

Supplementary Table S3. Table showing analyte concentrations (ng/mL) in diagnostic groups stratified by APOE ε4 carrier status.

|  | E4-Control | E4+Control | E4-mTBI | E4+mTBI | E4-PTSD | E4+PTSD | E4-mTBI+PTSD | E4+mTBI+PTSD |
| --- | --- | --- | --- | --- | --- | --- | --- | --- |
|  | Mean | SD | Mean | SD | Mean | SD | Mean | SD | Mean | SD | Mean | SD | Mean | SD | Mean | SD |
| 2-AG | 11.653 | 48.344 | 32.986 | 82.921 | 13.172 | 51.329 | 0.265 | 0.174 | 5.3068 | 24.024 | 0.2098 | 0.115 | 0.3095 | 0.1454 | 0.4201 | 0.2062 |
| 16:0 EA | 1.1466 | 0.4038 | 1.1957 | 0.4835 | 1.2271 | 0.2884 | 0.9822 | 0.2201 | 1.3767 | 0.4093 | 1.0514 | 0.222 | 1.0281 | 0.3467 | 1.1675 | 0.4209 |
| 16:1 EA | 1.3298 | 5.3457 | 2.5645 | 6.3773 | 1.2464 | 4.5252 | 0.0673 | 0.0322 | 0.6088 | 2.3604 | 0.087 | 0.0564 | 0.0846 | 0.0242 | 0.1582 | 0.0873 |
| 18:0 EA | 1.066 | 0.9829 | 0.9068 | 0.2881 | 0.8894 | 0.2532 | 0.9295 | 0.3696 | 1.3446 | 1.4468 | 0.8495 | 0.2788 | 0.9099 | 0.5476 | 0.9231 | 0.5141 |
| 18:1 EA | 5.237 | 3.1065 | 4.8108 | 1.5104 | 6.8034 | 4.5023 | 7.7144 | 7.5494 | 9.2418 | 16.783 | 5.4398 | 3.2514 | 12.956 | 23.752 | 5.9853 | 1.7053 |
| 18:2 EA | 5.6785 | 23.167 | 11.408 | 28.118 | 6.7121 | 22.77 | 0.4444 | 0.1881 | 2.4096 | 9.1105 | 0.4266 | 0.1936 | 0.4828 | 0.32 | 0.527 | 0.2325 |
| 20:4 EA | 0.2525 | 0.1004 | 0.1919 | 0.0816 | 0.2721 | 0.1184 | 0.241 | 0.0782 | 0.258 | 0.0908 | 0.249 | 0.1539 | 0.2056 | 0.0525 | 0.3396 | 0.1409 |
| 22:4 EA | 0.0757 | 0.0335 | 0.0699 | 0.0224 | 0.0957 | 0.0371 | 0.0744 | 0.0258 | 0.0857 | 0.0401 | 0.0768 | 0.0419 | 0.0615 | 0.0247 | 0.0856 | 0.0489 |
| 22:6 EA | 0.4006 | 1.876 | 0.0792 | 0.0373 | 0.1055 | 0.0458 | 0.0879 | 0.04 | 0.1144 | 0.0615 | 0.0868 | 0.0521 | 0.0952 | 0.0409 | 0.112 | 0.06 |
| 9(10)-DiHOME | 8.2345 | 4.2962 | 9.1378 | 6.6569 | 9.4541 | 4.8899 | 8.9933 | 5.9544 | 9.0432 | 3.7541 | 8.2763 | 3.3901 | 8.798 | 5.2444 | 5.8572 | 3.0052 |
| 9(10)EpOME | 0.8748 | 0.6579 | 0.8886 | 0.8315 | 0.9887 | 0.7697 | 0.9449 | 0.3638 | 1.1021 | 0.6098 | 0.7154 | 0.3828 | 1.105 | 0.7013 | 0.8433 | 0.4456 |
| 12(13)-DiHOME | 12.518 | 7.7186 | 13.977 | 10.242 | 13.814 | 6.1218 | 16.44 | 9.2137 | 13.415 | 7.8887 | 13.423 | 5.046 | 16.004 | 7.2252 | 11.731 | 5.5954 |
| 12(13)EpOME | 1.7888 | 1.241 | 2.168 | 1.4976 | 2.2272 | 1.3837 | 2.422 | 0.9829 | 2.4649 | 2.2603 | 2.3371 | 1.4733 | 3.1999 | 1.6546 | 1.3735 | 0.3282 |
| 9-HODE | 3.3875 | 1.1484 | 3.0248 | 1.0951 | 3.3109 | 1.4197 | 3.7427 | 0.6024 | 3.7739 | 1.6717 | 3.2935 | 1.2274 | 3.852 | 1.4467 | 2.4031 | 0.8654 |
| 13-HODE | 2.5367 | 0.9309 | 2.4323 | 0.9851 | 2.7252 | 1.3301 | 2.9222 | 0.545 | 3.0033 | 1.592 | 2.405 | 0.744 | 2.682 | 0.8945 | 1.7754 | 0.584 |
| 5-HETE | 0.4729 | 0.3375 | 0.2844 | 0.2186 | 0.5504 | 0.5235 | 0.4695 | 0.3022 | 0.5247 | 0.3401 | 0.5614 | 0.6247 | 0.5846 | 0.4012 | 0.7274 | 0.4011 |
| 8-HETE | 0.3691 | 0.2854 | 0.2335 | 0.1617 | 0.2729 | 0.191 | 0.3277 | 0.2388 | 0.4597 | 0.5859 | 0.2711 | 0.2503 | 0.3129 | 0.1983 | 0.4685 | 0.1509 |
| 11-HETE | 0.305 | 0.5076 | 0.114 | 0.0721 | 0.1146 | 0.0819 | 0.132 | 0.0965 | 0.1523 | 0.0743 | 0.1178 | 0.1027 | 0.1233 | 0.076 | 0.2349 | 0.0667 |
| 15-HETE | 0.2225 | 0.1472 | 0.1666 | 0.1299 | 0.205 | 0.1842 | 0.2351 | 0.1502 | 0.2744 | 0.1531 | 0.2075 | 0.1831 | 0.249 | 0.1818 | 0.2926 | 0.1211 |
| 19(S)-HETE | 0.0343 | 0.0238 | 0.0267 | 0.0199 | 0.0328 | 0.0359 | 0.0387 | 0.0188 | 0.0437 | 0.0293 | 0.0269 | 0.021 | 0.0418 | 0.0442 | 0.075 | 0.0504 |
| 8(9)-DiHET | 0.0279 | 0.0233 | 0.0242 | 0.0181 | 0.0399 | 0.0625 | 0.0356 | 0.0202 | 0.0495 | 0.05 | 0.0267 | 0.0255 | 0.089 | 0.0941 | 0.0855 | 0.0678 |
| 11(12)-DiHET | 0.2202 | 0.0765 | 0.1858 | 0.06 | 0.228 | 0.152 | 0.2178 | 0.0544 | 0.3172 | 0.1354 | 0.2053 | 0.0932 | 0.2911 | 0.2263 | 0.4743 | 0.3153 |
| 14(15)-DiHET | 0.3544 | 0.1265 | 0.3226 | 0.0861 | 0.3809 | 0.2433 | 0.3642 | 0.0661 | 0.4847 | 0.1854 | 0.3375 | 0.1198 | 0.45 | 0.3371 | 0.652 | 0.3875 |
| 8(9)EET | 0.0145 | 0.0138 | 0.0091 | 0.0122 | 0.0203 | 0.0275 | 0.0171 | 0.0148 | 0.0275 | 0.0237 | 0.0116 | 0.0141 | 0.0205 | 0.0318 | 0.0348 | 0.0309 |
